# Salmonella Effector SpvC Targets SEC23B of Intestinal Epithelial Cells to Resist Gasdermin D-Mediated Protection Against Systemic Infection

**DOI:** 10.3390/microorganisms14051148

**Published:** 2026-05-19

**Authors:** Liting Zhou, Yan Yang, Li Kang, Jiayi You, Ye Wang, Ailing Xu, Guangmin Tu, Rui Huang, Zhengyu Zhou, Minghui Li, Shuyan Wu

**Affiliations:** 1 School of Basic Medical Science, Suzhou Medical College of Soochow University, Suzhou 215123, China; 15043200271@163.com (L.Z.); 13587169217@163.com (Y.Y.); kangli20010225@gmail.com (L.K.); youjiayi97@163.com (J.Y.); 19951035531@163.com (Y.W.); xai20117@163.com (A.X.); 18185189689@163.com (G.T.); hruisdm@163.com (R.H.); 2 Center of Clinical Laboratory, The Fourth Affiliated Hospital of Soochow University, Suzhou 215123, China; 3 MOE Key Laboratory of Geriatric Diseases and Immunology, Suzhou Key Laboratory of Pathogen Bioscience and Anti-Infective Medicine, Suzhou Medical College of Soochow University, Suzhou 215123, China; 4 Laboratory Animal Center, Suzhou Medical College of Soochow University, Suzhou 215123, China; zacharyzhou@suda.edu.cn

**Keywords:** salmonella effector SpvC, GSDMD, intestinal epithelial barrier, SEC23B

## Abstract

Salmonella infects a wide range of hosts, causing gastroenteritis or systemic infection in humans and animals, highlighting the urgent need for a deeper understanding of its pathogenesis. SpvC, a critical virulence determinant of salmonella, facilitates bacterial dissemination. Gasdermin D (GSDMD) is the only gasdermin known to protect mice against acute *Salmonella* enteritis. Our preliminary findings indicated that SpvC counteracts GSDMD-mediated antibacterial effects to enhance bacterial dissemination, although its functional relevance to epithelial-derived GSDMD and the underlying mechanisms remain unclear. To address this, *Gsdmd*^−/−^ C57BL/6J and wild-type mice were infected with *Salmonella* Typhimurium (*S*. Typhimurium) wild-type strain and *spvC* deletion mutant. Our results demonstrate that SpvC compromises intestinal epithelial barrier integrity, overcoming GSDMD-mediated protection against systemic infection. Specifically, through bioinformatics analysis, LC-MS/MS, and in vivo experiments with Caco-2 cell monolayers and site-directed *spvC* mutants, we identified SEC23B as a novel target of SpvC. This interaction disrupts the intestinal epithelial barrier through the autophagy–pyroptosis pathway. This study identifies SEC23B as a unique cellular target of SpvC involved in GSDMD activation during *S*. Typhimurium systemic infection. It also reveals a novel mechanism by which Salmonella evades host defense mechanisms.

## 1. Introduction

*Salmonella* Typhimurium (*S*. Typhimurium), as an important foodborne pathogen, has a broad range of hosts and causes gastroenteritis or systemic infection in humans and animals. The frequent mutations of salmonella serotypes and the rising prevalence of antibiotic-resistant strains have posed substantial challenges to clinical treatment [1]. *S*. Typhimurium also serves as a valuable model organism for studying the mechanisms underlying host–pathogen interactions [2]. *S*. Typhimurium colonizes the gut lumen, breaches the intestinal epithelial barrier, and can disseminate to systemic organs, causing life-threatening infections in immunocompromised individuals. As a facultative intracellular bacterium that coexists and coevolves with its human host, *S*. Typhimurium has developed various strategies to sustain prolonged infection. The delivery of effectors into host cells plays a critical role in its pathogenesis. One such effector, SpvC, has been shown to alleviate intestinal inflammation, thereby facilitating bacterial dissemination [3]. Gasdermin D (GSDMD), a critical mediator of innate immune response, significantly influences the progression and outcome of infection. Our previous study revealed that the pathogenesis of *S*. Typhimurium SpvC is closely related to host GSDMD [4]. However, the specific host cellular targets and regulatory mechanisms underlying SpvC–host interactions remain largely unexplored.

Gasdermins are composed of six paralogous genes with high expression in the gastrointestinal tract [5]. Except for PJVK, the N terminus of the gasdermin proteins can oligomerize and insert into the plasma membrane [6,7]. Research findings on the role of GSDMD in intestinal inflammation have yielded contradictory results. In the DSS-induced colitis model, deletion of *Gsdmd* either aggravated intestinal inflammation or displayed a protective phenotype compared to wild-type mice [8,9]. Furthermore, conditional deletion of the upstream regulators of GSDMD, such as NEK or TRIM2, in macrophages resulted in protection against intestinal inflammation, indicating a functional diversity of GSDMD across different cell types [10,11]. We previously demonstrated that GSDMD orchestrates macrophage–neutrophil cooperation to prevent excessive phagocyte infiltration and limit bacterial dissemination in response to *S*. Typhimurium effector SpvC [12]. Evidence has also shown that GSDMD is the only gasdermin that enhances mucosal barrier integrity against salmonella gut infection [13]. It remains to be clarified how SpvC overcomes the host defense mediated by GSDMD, allowing *S*. Typhimurium to spread beyond the intestinal barrier.

The mechanical barrier formed by intestinal epithelial cells (IECs) and their intercellular junctions serves as a crucial defense against *S*. Typhimurium invasion [14]. GSDMD-associated pyroptosis enables the shedding of *S*. Typhimurium-infected IECs, with neighboring cells rearranging to sustain epithelial integrity [15,16]. Caspase-7 repairs GSDMD pores and maintains cell integrity by activating acid sphingomyelinase [17]. Nevertheless, it remains unclear whether the *S*. Typhimurium effector SpvC impairs intestinal mucosa integrity via GSDMD-mediated pyroptosis pathways.

SpvC, a key virulence factor of *S*. Typhimurium, encodes phosphothreonine lyase that inactivates dual-phosphorylated host mitogen-activated protein kinases (MAPK) [3]. We previously observed that depleting the phosphothreonine lyase activity of SpvC is insufficient to reverse its effects on host cell pyroptosis [4], suggesting that SpvC may possess additional functions that counteract host antimicrobial defenses. Here, using bioinformatic analysis and liquid chromatography–tandem mass spectrometry (LC-MS/MS), we report that SpvC targets host SEC23B, thereby subverting the autophagy–pyroptosis pathway and disrupting the intestinal epithelial barrier to exacerbating systemic *S*. Typhimurium infection.

## 2. Materials and Methods

The sources and identifiers of all key reagents and resources are listed in Table S1.

### 2.1. Bacterial Strains

*S*. Typhimurium wild-type strain SL1344 (STM-WT) was kindly supplied by Professor Shao Feng (National Institute of Biological Sciences, Beijing, China) [18]. STM-WT, *spvC* deletion mutant (STM-Δ*spvC*), *spvC* complemented strain (STM-Δ*spvC*/p*spvC*) and *spvC* site-directed mutant were constructed as previously described [4]. STM-Δ*spvC* carrying empty pBAD (STM-Δ*spvC*/pBAD) or pBAD for His-tagged SpvC expression (STM-Δ*spvC*/*spvC*:His) were constructed in this study. The primers used for bacterial strain construction are listed in Table S2.

### 2.2. Mice and Ethics Statement

Wild-type C57BL/6J mice and *Gsdmd*^−/−^ mice were purchased from GemPharmatech (San Diego, CA, USA). Female mice aged 6~8 weeks old were used in the study. Mice were fed locally on a standard chow diet and housed in a temperature- and light-regulated room at the specific pathogen-free (SPF) facility.

### 2.3. Cell Lines

Caco-2, J774A.1, HeLa, and HEK293T cells were routinely cultured in Dulbecco’s modified Eagle medium (DMEM) supplemented with 10% (*v*/*v*) fetal bovine serum at 37 °C in a humidified incubator containing 5% CO_2_.

### 2.4. Bacterial Culture and Infections

*S*. Typhimurium was grown at 37 °C in Luria–Bertani (LB) broth overnight. The STM-Δ*spvC*/p*spvC*, STM-Δ*spvC*/pBAD and STM-Δ*spvC*/*spvC*:His were cultured in the medium with 100 μg/mL ampicillin. On the day of infection, *S*. Typhimurium was subcultured 1:100 in fresh LB broth to log phase. STM-Δ*spvC*/p*spvC*, STM-Δ*spvC*/pBAD and STM-Δ*spvC*/*spvC*:His were supplemented with 0.2% L-arabinose. Bacteria were then washed three times in PBS. The optical density of bacteria was determined by spectrophotometry at 600 nm with viable plate counts prior to infection.

For in vivo infection, a streptomycin-pretreated murine model was established as previously described [4]. Mice were fasted for 4 h before oral administration of streptomycin (20 mg/each in 200 μL). After 20 h, wild-type and *Gsdmd*^−/−^ C57BL/6J mice were orally infected with 5 × 10^7^ STM-WT or STM-Δ*spvC* (in 100 μL). Mice were euthanized using CO_2_ asphyxiation at the indicated time points.

For in vitro infection, Caco-2 cells (3 × 10^5^/well), J774A.1 cells (8 × 10^5^/well) and HeLa cells (5 × 10^5^/well) were seeded in 12-well plates. The bacterial suspension was added to cultured cells at the multiplicity of infection (MOI) described in the figure legends. Fresh medium containing 100 μg/mL amikacin was added to kill the extracellular bacteria at 1 hpi. After 2 h, cells were washed with PBS and incubated in medium containing 10 μg/mL amikacin to limit replication of extracellular bacteria. Cells were pretreated with DSF (30 μM) 1 h before infection [19].

### 2.5. Bacterial Burden Measurement

From each group, four samples of spleen and liver were collected and homogenized. CFU of the bacteria were determined by serial-dilution plating on salmonella–shigella agar plates. Colonies were calculated per gram of tissue after 16 h of incubation [16].

### 2.6. Histopathological Analysis and Immunofluorescence Staining

From each group, five samples of liver, spleen and ceca samples were fixed in 10% formaldehyde solution and embedded in paraffin. For histopathological analysis, sections (5 μm) were stained with hematoxylin–eosin and observed under a light microscope (Olympus). Total inflammatory scores (0–20) were evaluated based on neutrophil infiltration, fibrin deposition, submucosal neutrophil margination, submucosal edema, epithelial necrosis, and epithelial ulceration, following published methods [20].

For immunofluorescence staining, ceca sections (6 μm) were deparaffinized and rehydrated for antigen retrieval. After blocking with 3% BSA for 30 min at room temperature, sections were incubated overnight at 4 °C with primary antibodies, followed by staining with Cy3-labeled secondary antibodies. Images were photographed using a Nikon microscope (ECLIPSE, Ts2R-FL, Tokyo, Japan).

### 2.7. Transmission Electron Microscopy

Sample processing for transmission electron microscopy was carried out in the School of Biology and Basic Medical Science, Medical College of Soochow University. Briefly, ceca samples were cut into 1 mm^3^ and fixed using ice-cold 4% glutaraldehyde for at least 4 h. Fixed samples were washed twice using 0.1 M phosphate buffer for 15 min at room temperature. Post-fixing was done using 1% OsO4 for 1 h. Samples were stored in an ethanol series and embedded in epoxy resin. Ultra-thin sections were stained and observed using a 120 kV transmission electron microscope (HT7700, Hitachi, Tokyo, Japan).

### 2.8. Intestinal Epithelial Barrier Function Assay In Vitro

Caco-2 cells were seeded in 12-well Transwell inserts at a density of 3 × 10^5^ cells/well, with medium changed every other day. TEER values were measured by a Millicells Voltmeter (Millipore, Burlington, MA, USA) according to the manufacturer’s protocol. Monolayers with TEER values above 200 Ω/cm^2^ (±10) indicate that the cells were successfully established [21].

For Western blotting analysis, detergent-insoluble (membrane) and detergent-soluble (cytosolic) proteins were isolated using a Membrane and Cytosol Protein Extraction Kit (Beyotime, Shanghai, China).

### 2.9. Cell Transfection

HEK293T cells transfected with plasmids were conducted using Lipofectamine 3000 (Thermo Fisher Scientific, Wilmington, DE, USA) following the manufacturer’s protocol. ExFect transfection (Thermo Fisher Scientific, Wilmington, DE, USA) reagent was used for siRNA transfection in HeLa cells according to the manufacturer’s instructions.

### 2.10. Western Blotting Analysis

Proteins were extracted using RIPA buffer containing protease inhibitors and phosphatase inhibitors. Lysates were separated on an 8–15% SDS-polyacrylamide gel, transferred onto PVDF membranes, and blocked for 1 h in 5% skimmed milk. Membranes were incubated in primary antibodies overnight at 4 °C, and then washed and incubated for 1 h with HRP-conjugated secondary antibodies. After washing with TBST (3X), the blots were developed using an enhanced chemiluminescence system (Tannon). Densitometric analysis of Western blots was done using ImageJ software (ImageJ 1.45S).

### 2.11. In Silico Prediction of Binding Affinity and the Effects of Mutations on SpvC–Protein Complexes

For SpvC and 30 human proteins, we initially obtained the corresponding UniProt IDs, sequence lengths, PDB crystal structures, and AlphaFold2 predicted structure annotations from the UniProt database [22]. Using the PDB IDs retrieved, we accessed the Protein Data Bank (PDB) to acquire the protein structure files. Additionally, we utilized the InterPro database to obtain the Pfam domains of the proteins [23]. We prioritized experimentally determined crystal structures based on two criteria to ensure relatively complete and high-quality protein structures: high resolution and high structure coverage and/or inclusion of important functional domains. In cases where an experimentally determined crystal structure was not available, we used the AlphaFold2 predicted structure [24,25]. For predicted structures, we selectively removed large loop regions or disordered regions at the N- and C-termini that had low prediction accuracy. Our final selection aimed to include functional domains as comprehensively as possible. Table S3 provides the basic information for all proteins and the structural ranges ultimately used.

Subsequently, we utilized the ZDOCK tool (v3.0.2) [26] to perform docking and generate SpvC–protein complex structures for each of the 30 protein targets. ZDOCK conducts a comprehensive rigid-body search to explore potential docking orientations between two proteins, incorporating performance optimizations and a novel pairwise statistical energy potential to enhance accuracy. We configured the docking runs to generate 2000 conformations per run. ZDOCK provides a ZDOCK score, an internal scoring function estimating the likelihood of interaction between two proteins. The conformations are ranked based on this score, with higher ZDOCK scores indicating more stable docking conformations and a higher probability of representing the true complex structure. Based on the ZDOCK scores, we selected the top ten best-scoring conformations for further analysis. The binding energy (∆G, kcal mol^−1^) of these selected conformations was calculated using the AnalyseComplex module of FoldX (FoldX4) [27].

MutaBind2 (MutaBind v2.0) [28] and BeAtMuSiC (BeAtMuSiC v1.0) [29], two well-established machine learning approaches, were employed to identify mutations in SpvC that influence its binding with SEC23B by predicting changes in binding affinity (∆∆G, kcal mol^−1^) upon mutations. Additionally, we used ClusPro (ClusPro v2.0) [30] and HDOCK (HDOCK v1.1) [31] to generate the top ten highest-scoring conformations for the SpvC-SEC23B complex. These conformations were then analyzed to calculate ∆∆G values for further validation. Initially, we identified all interface residues in SpvC across the top ten best-scoring conformations obtained from ZDOCK. Interface residues were defined as those where any heavy atom from different protein chains was within an inter-atomic distance of less than 6 Å. Subsequently, we calculated the ∆∆G values for all possible mutations at these interface sites across the 30 top-scoring conformations, with 10 conformations generated by each of the tools—ZDOCK (ZDOCK 3.0.2), ClusPro, and HDOCK—using BeAtMuSiC. Finally, we also calculated the ∆∆G for alanine mutations at these interface sites across the same set of conformations using MutaBind2.

### 2.12. Immunoprecipitation Assays

Resuspend agarose beads in a vortex mixer, then add 20 μL beads to 1 mL protein sample and mix for 30 min at 4 °C. Centrifuge at 2500 rpm for 5 min at 4 °C, transfer the supernatant to a new ep tube. Add 45 μL of agarose beads and mix at 4 °C for 1 h. Add 7 μL of the His-Tag Monoclonal antibody (66005-1, Proteintech, Rosemont, IL, USA) and continue mixing at 4 °C overnight. Centrifuge at 2500 rpm for 5 min at 4 °C, discard the supernatant, wash with pre-cooled PBS 4 times, add 5× loading buffer according to the ratio, and boil at 100 °C for 5 min, then centrifuge the supernatant for subsequent Western blotting.

For SpvC interactome analysis, two 15 cm dishes of 293T cells transfected with EGFP-HA-SpvC or EGFP-HA-N1 were immunoprecipitated with anti-HA beads. Proteins were denatured by adding SDS loading buffer and boiling for 5 min, followed by LC-MS/MS determination.

### 2.13. Statistical Analysis

All data are presented as mean ± SD. Statistical analyses were performed using GraphPad Prism 9.0 (GraphPad Software, San Diego, CA, USA). Normality of data distribution was assessed using the Shapiro–Wilk test, and homogeneity of variances was evaluated by Levene’s test. For comparisons between two groups, the unpaired two-tailed Student’s *t*-test was applied. For comparisons involving three or more groups, one-way ANOVA followed by Tukey’s post hoc test for multiple comparisons was used. When two independent variables were involved, two-way ANOVA with Šidák’s or Bonferroni’s post hoc test was performed. A value of *p* < 0.05 was considered statistically significant.

## 3. Results

### 3.1. The Bacterial Effector SpvC and Host GSDMD Jointly Determine the Outcome of Salmonella Infection

**Figure 1:**
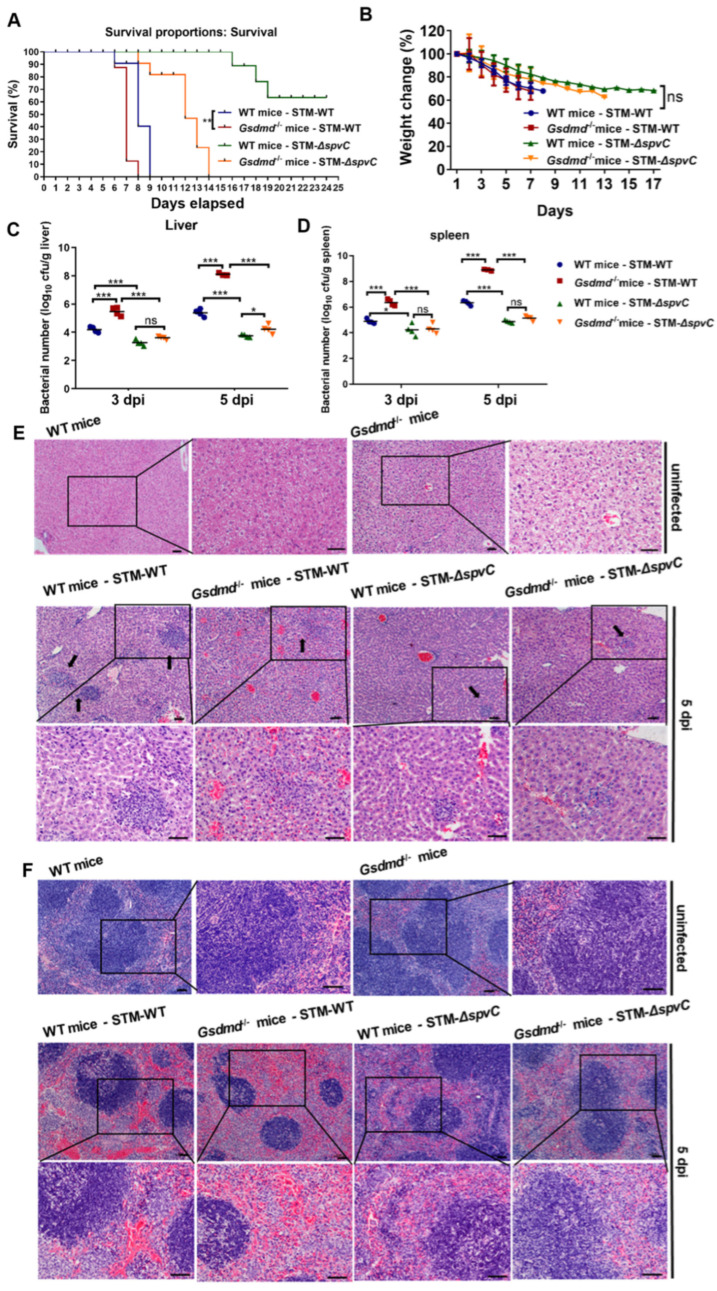
The bacterial effector SpvC and host GSDMD jointly determine the outcome of salmonella infection. C57BL/6J and *Gsdmd*^−/−^ mice were infected orally with 5 × 10^7^ colony-forming units (CFU) of either STM-WT or STM-Δ*spvC* after being pretreated with streptomycin. (**A**) Survival. (**B**) Body weight change, *n* = 10. (**C**,**D**) The bacterial loads of livers (**C**) and spleens (**D**), *n* = 4. (**E**,**F**) Histopathological analysis of the livers (**E**) and spleens (**F**) in the uninfected group or at 5 dpi, *n* = 5. Black arrows indicated infiltration of inflammatory cells. Scale bars, 50 µm. Data were compared by two-way ANOVA. Values are expressed as the means ± SD. Each dot represents data from one animal. Statistically significant differences are indicated. * *p* < 0.05, ** *p* < 0.01, *** *p* < 0.001, ns: not significant. Data were from three biological replicates.

Recent research has identified GSDMD as the sole gasdermin that protects against acute salmonella gut infection in mice [13]. To assess the potential role of GSDMD in the host defense against *S*. Typhimurium infection mediated by SpvC, we orally infected the streptomycin-pretreated WT mice and *Gsdmd*^−/−^ mice with either STM-WT or STM-Δ*spvC*, respectively. Mortality and body weight change were monitored during the course of infection. The susceptibility of *Gsdmd*^−/−^ mice was comparable to that of WT mice, both in the STM-WT and STM-Δ*spvC* infected groups. All *Gsdmd*^−/−^ mice gavaged with STM-WT succumbed to infection within 8 days post-infection (dpi), while during the same time span, 40% of the WT mice infected with STM-WT were still alive. In STM-Δ*spvC*-infected groups, *Gsdmd*^−/−^ mice succumbed to infection within 14 days, whereas 60% of the WT mice survived beyond 19 dpi (Figure 1A). These results highlighted the critical role of GSDMD in early innate immune responses, which are essential for controlling the pathogen and ultimately determining the outcome of the infection. All mice showed a gradual decrease in body weight without any significant difference among these groups (Figure 1B). To further investigate the role of GSDMD in systemic infection mediated by *S*. Typhimurium SpvC, livers and spleens were harvested at 3 dpi and 5 dpi to quantify the bacterial burden. As the infection progressed, increased bacterial loads were observed in the livers and spleens of mice infected with *S.* Typhimurium. The absence of GSDMD resulted in significantly higher bacterial loads in the livers and spleens of mice infected with STM-WT. Deletion of *spvC* in *S.* Typhimurium not only led to decreased bacterial loads in spleen and liver but also reduced or even eliminated the difference in bacterial loads between WT and *Gsdmd*^−/−^ mice (Figure 1C,D). The protective effect of GSDMD was highlighted by pathological analysis. At 1 dpi, no obvious lesions were detected in the livers and spleens among all groups. However, progressive pathological lesions were observed from 3 dpi to 5 dpi (Figure 1E,F and Figure S1A,B). In STM-WT-infected mice at 5 dpi, GSDMD deficiency exacerbated inflammatory cell infiltration and hemorrhage in livers (Figure 1E). Spleens from STM-WT-infected mice demonstrated an expansion of the red pulps and atrophy of white pulps due to the lack of GSDMD (Figure 1F). STM-Δ*spvC* modestly affected the pathological injury in both livers and spleens. The aforementioned results suggest GSDMD plays a critical role in host defense against *S*. Typhimurium infection by limiting systemic bacterial dissemination and mitigating tissue damage, particularly in the context of SpvC-dependent virulence.

### 3.2. Salmonella Effector SpvC Overcomes GSDMD-Mediated Protection from Intestinal Epithelial Barrier of Mice

**Figure 2:**
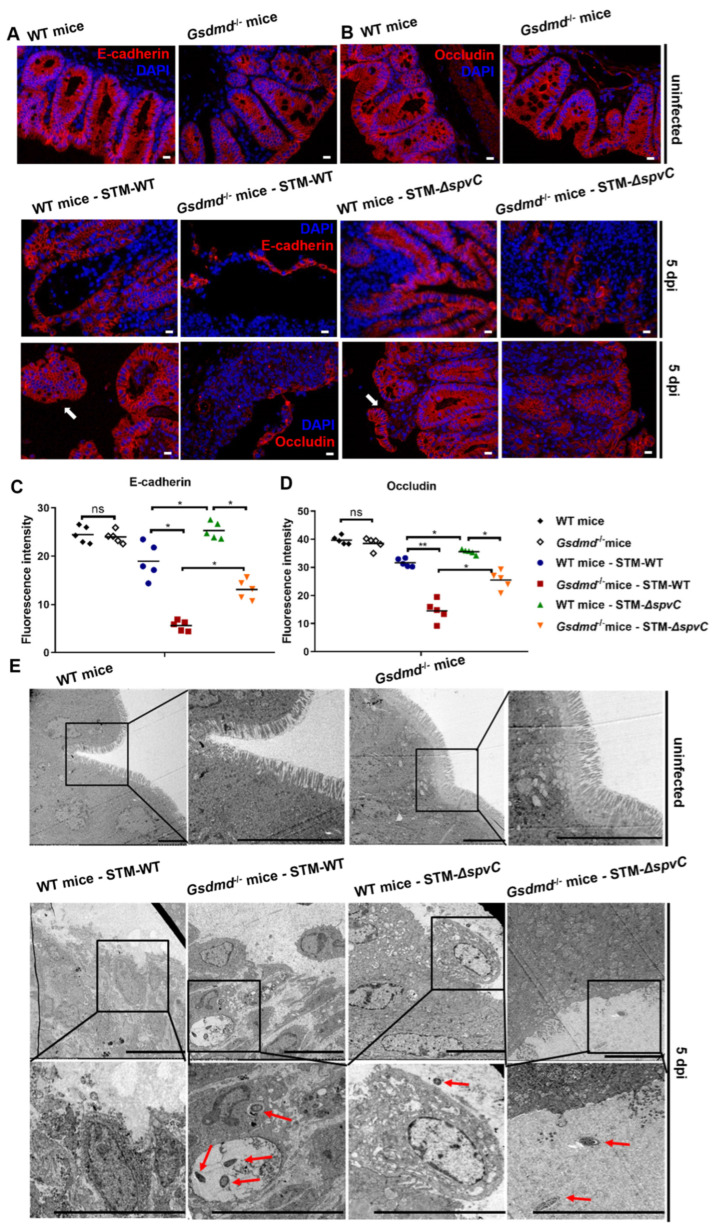
Salmonella effector SpvC overcomes GSDMD-mediated protection of the intestinal epithelial barrier of mice. C57BL/6J and *Gsdmd*^−/−^ mice were infected orally with 5 × 10^7^ CFU of either STM-WT or STM-Δ*spvC* after being pretreated with streptomycin, *n* = 5. Representative images of immunofluorescence staining for E-cadherin (**A**) and Occludin (**B**) on frozen sections of ceca in the uninfected group or at 5 dpi, *n* = 5. White arrows indicated disruption of the epithelial barrier. Scale bars, 50 μm. (**C**,**D**) Quantification of fluorescence intensity. Each dot represents data from one animal. Data were compared by two-way ANOVA. (**E**) TEM images of the ultrastructure of intestinal epithelial cells in the uninfected group or at 5 dpi. Red arrows indicated bacteria. Scale bars, 5 μm. Data were compared by two-way ANOVA. Values are expressed as the means ± SD. * *p* < 0.05, ** *p* < 0.01, ns: not significant. Data were from three biological replicates.

In the case of *S*. Typhimurium infection, the intestinal tract is the main theater of the host–pathogen tug-of-war [32]. To assess intestinal injury in mice infected with *S*. Typhimurium, hematoxylin–eosin (HE) staining was performed. At 3 dpi in STM-WT-infected mice, GSDMD was found to protect against intestinal pathology, as evidenced by the disruption of IECs observed in *Gsdmd*^−/−^ mice. The absence of *spvC* led to an increased influx of inflammatory cells into the lamina propria and submucosa of the ceca, supporting the notion that SpvC ameliorates intestinal inflammation during *S*. Typhimurium infection [3,4]. Notably, these differences in pathological manifestations between WT and *Gsdmd*^−/−^ mice were significantly reduced following *spvC* ablation, further supporting its role in modulating intestinal inflammation (Figure S2A,B). These results indicated that SpvC counteracts GSDMD-mediated protection from intestinal injury of mice. The mechanical barrier formed by IECs and their intercellular connections is considered a crucial defense against *S*. Typhimurium infection. To evaluate the impact of SpvC on intestinal epithelial barrier and its relevance to GSDMD, we used immunofluorescence labeling to visualize the expression and distribution of barrier-related proteins E-cadherin and Occludin. Results unraveled a progressive collapse of the intestinal epithelial barrier as the infection persisted. In mice infected with *S*. Typhimurium carrying *spvC*, IECs labeled with E-cadherin or Occludin were shed at 1 dpi, suggesting that SpvC impairs the integrity of the epithelial barrier (Figure S2C,D). At 3 dpi, WT mice displayed clearer and more intact staining for E-cadherin and Occludin compared to *Gsdmd*^−/−^ mice. Consistent with the histopathological findings, fewer crypts were observed in *Gsdmd*^−/−^ mice than in WT mice (Figure S2C,D). These observations highlight the role of GSDMD in maintaining the intestinal epithelial barrier in response to *S*. Typhimurium infection. At 5 dpi, STM-WT infected WT mice exhibited a significant loss of IECs and severe disruption of the intestinal epithelial barrier compared to *Gsdmd*^−/−^ mice. However, in STM-Δ*spvC*-infected mice, GSDMD did not play a remarkable role in preserving the intestinal epithelial barrier (Figure 2A–D). Together, these findings suggest that host GSDMD mitigates SpvC-mediated disruption of the intestinal epithelial barrier during *S*. Typhimurium infection.

We further visualized the ultrastructure of ceca using a transmission electron microscope (TEM). In *Gsdmd*^−/−^ mice infected with STM-WT, the brush border was partially disorganized and lesions exacerbated at 5 dpi. Compared to WT mice, *gsdmd*^−/−^ mice displayed more severe anomalies in brush border structures and a greater spread of bacteria into the lamina propria was observed in STM-WT-infected groups. In contrast, STM-Δ*spvC*-infected groups showed reduced bacterial colonization overall. Furthermore, the differences in bacterial colonization between WT and *Gsdmd*^−/−^ mice were reduced in the absence of SpvC (Figure 2E). These data support the conclusion that SpvC acts as a virulence determinant by enhancing *S*. Typhimurium adhesion and colonization in IECs. Meanwhile, GSDMD, as an essential component of the immune system, probably preserves the integrity of the epithelial barrier to counteract SpvC-mediated *S*. Typhimurium distribution.

### 3.3. SpvC Physically Interacts with SEC23B in Epithelial Cells

**Figure 3:**
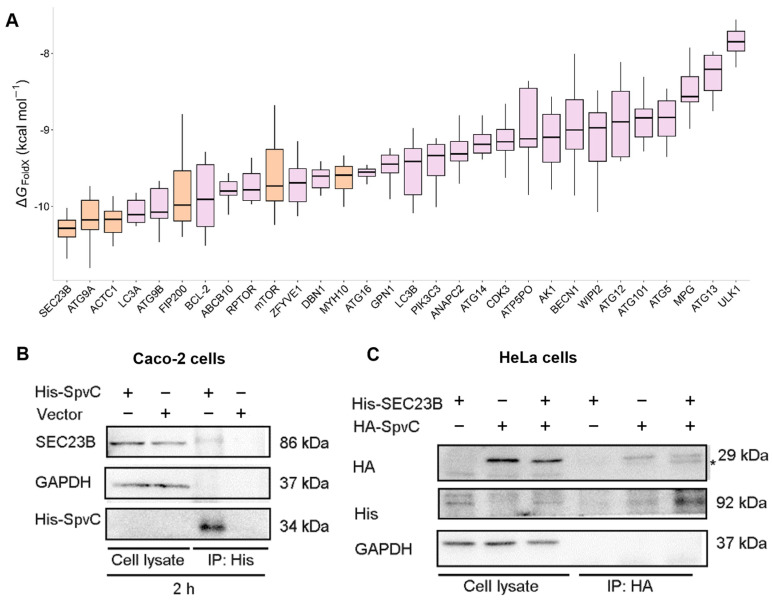
SpvC physically interacts with SEC23B in epithelial cells. (**A**) Binding affinity (∆G, kcal mol^−1^) of SpvC with each of the 30 protein targets was calculated using the AnalyseComplex module of FoldX. This analysis was performed using the top ten best-scoring conformations for each complex generated by ZDOCK. The *X*-axis represents gene symbol, and the *Y*-axis represents binding affinity (ΔG). (**B**) Caco-2 cells were infected with STM-Δ*spvC* carrying an empty vector or a vector expressing SpvC: His at an MOI of 100 for 2 hpi. (**C**) HeLa cells were transfected with pcDNA3.1-SEC23B-6*His and/or pEGFP -SpvC-HA. * indicated the non-specific band. His/HA-tagged SpvC were immunoprecipitated from cell lysates and assessed for their ability to bind endogenous or exogenous SEC23B.

Considering that SpvC impedes the host protective function of GSDMD, we hypothesized that a host interaction partner (or partners) of SpvC might be responsible for GSDMD activation. To investigate this, we performed LC-MS/MS analysis to compare protein components enriched in EGFP-HA-SpvC or EGFP-HA-N1-transfected cells. The proteins identified as unique or highly enriched in the HA-SpvC samples are shown in Table 1. Since SpvC was previously found to inhibit autophagosome formation in host cells [33], 18 proteins [34] closely associated with this process were also selected for bioinformatic analysis. Due to the differences in the conformations generated by docking simulations, we selected the top 10 highest-scoring structures for further study to derive more reliable results than those obtained from a single conformation. The binding affinity (∆G, kcal mol^−1^) of SpvC with each of the 30 protein targets (12 proteins screened by LC-MS/MS and 18 proteins related to autophagy) was calculated, and the interaction strength across different proteins is presented in Figure 3A. More negative ∆G values indicated stronger binding. The protein with the strongest binding to SpvC was SEC23B. Next, we tested whether SpvC: His, translocated by *S*. Typhimurium, could interact with the top candidate proteins identified through bioinformatics analysis and proteomic profiling. The results showed SpvC interacts with host proteins SEC23B and ACTC1, while showing no detectable binding to mTOR, FIP200, or MYH10 in *S*. Typhimurium-infected Caco-2 cells (Figure 3B and Figure S3A,B). This upward shift on SDS-PAGE suggests that the interaction with the bacterial effector SpvC directly induces or is concomitant with a post-translational modification (PTM) of SEC23B. This SpvC-induced PTM would functionally alter SEC23B’s role in vesicular transport, potentially hijacking this process to prevent the delivery of antimicrobial cargo to degradative compartments such as autolysosomes [35]. The functional consequences of SpvC binding to ACTC1 in host defense will be reported separately. Additionally, SpvC expressed in HeLa cells was verified to bind with exogenous SEC23B, providing further evidence that SpvC could interact with both endogenous and exogenous SEC23B (Figure 3C).

### 3.4. SpvC Targets SEC23B to Restrict GSDMD-Mediated Host Defense Through Autophagy–Pyroptosis Pathway

**Figure 4:**
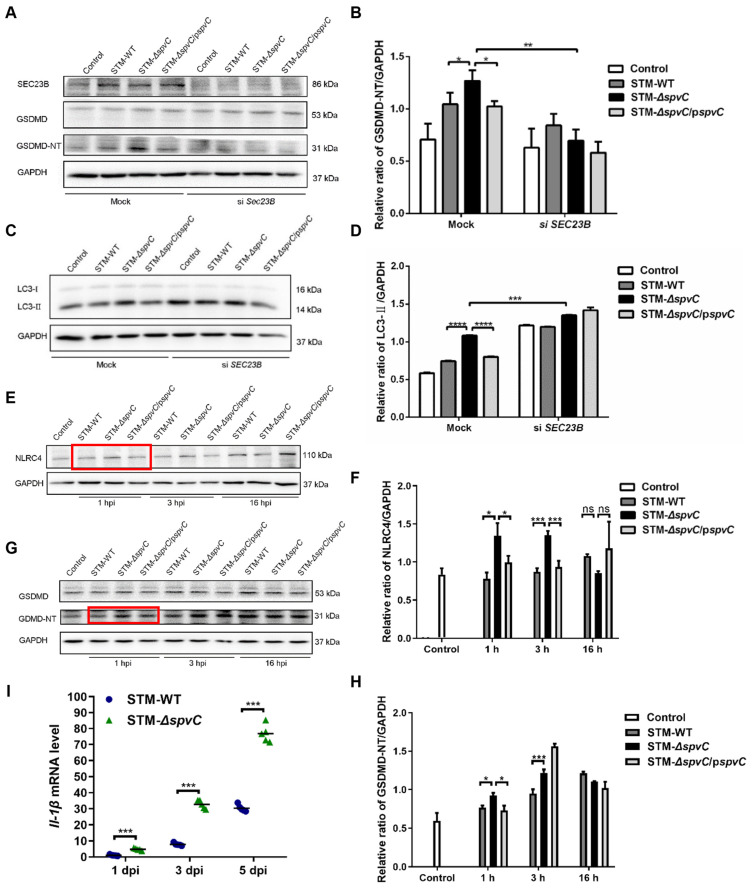
SpvC targets SEC23B to restrict GSDMD-mediated host defense through the autophagy–pyroptosis pathway. (**A**) HeLa cells were transfected with either a non-targeting siRNA oligo (Mock) or 30 nM SEC23B siRNA oligos and infected with the indicated *S*. Typhimurium strains at an MOI of 100. SEC23B, and GSDMD/GSDMD-NT were analyzed by Western blotting at 16 hpi. (**C**) LC3-I/II were analyzed by Western blotting at 2 hpi. Caco-2 cells were co-cultured with the indicated *S*. Typhimurium strains at an MOI of 100 for 1 h, 3 h and 16 h. NLRC4 (**E**) and GSDMD/GSDMD-NT (**G**) were analyzed by Western blotting. Red boxes indicate SpvC inhibits the protein levels of NLRC4 and GSDMD-NT. (**B**,**D**,**F**,**H**) Quantification of Western blotting analysis on the left. (**I**) C57BL/6J mice were pretreated with streptomycin and then orally infected with 5 × 10^7^ CFU of either STM-WT or STM-Δ*spvC*, *n* = 5. The transcriptional levels of *Il-1β* in cecal tissues were assessed by RT-qPCR at 1, 3 and 5 dpi. Each dot represents data from one animal. Data were compared by one-way ANOVA. Values are expressed as the means ± SD, and statistically significant differences are indicated. * *p* < 0.05, ** *p* < 0.01, *** *p* < 0.001, **** *p* < 0.0001, ns: not significant. Data were from three biological replicates.

To further investigate the role of SEC23B in GSDMD-mediated host defense against *S*. Typhimurium infection, HeLa cells were treated with siRNA to knockdown *SEC23B* and then co-cultured with STM-WT, STM-Δ*spvC*, and STM-Δ*spvC*/p*spvC*. Western blotting showed that much more GSDMD-NT was determined in STM-Δ*spvC*-infected groups than those infected with *S*. Typhimurium strains carrying *spvC* at 16 hpi. After *si*SEC23B treatment, the levels of GSDMD-NT significantly decreased (Figure 4A,B). These results confirm that the interaction between SpvC and SEC23B is essential for impairing GSDMD-mediated host defense.

We previously reported that SpvC inhibits pyroptosis to aggravate *S*. Typhimurium systemic infection in mice [4]. GSDMD was identified as the executor of pyroptosis [36]. Our previous study also demonstrated that salmonella *spvC* gene inhibits the autophagy of host cells and suppresses the activation of inflammasomes [33]. In the present study, we observed that knocking down *SEC23B* significantly increased the protein level of LC3-II in human cervical carcinoma epithelial HeLa cells infected with *S*. Typhimurium harboring *spvC* (Figure 4B,C), indicating that SpvC impairs autophagosome formation in an SEC23B-dependent manner. Emerging evidence highlights the intricate interplay between autophagy and the inflammasome in host defense [37]. Notably, NLRC4 has been shown to play a unique role in maintaining epithelial barrier integrity by driving the expulsion of pyroptotic IECs [38]. To investigate the role of the NLRC4-GSDMD axis in this pathogen–host conflict, we monitored the protein levels of NLRC4 and GSDMD in *S*. Typhimurium-infected Caco-2 cells. Western blot analysis revealed that deletion of *spvC* resulted in increased levels of NLRC4 during the early stage of infection. Interestingly, this trend was reversed at 16 h hpi, with NLRC4 levels decreasing in the STM-Δ*spvC*-infected group (Figure 4E,F). Correspondingly, we found an elevated abundance of GSDMD-N terminal (GSDMD-NT) in STM-Δ*spvC*-infected Caco-2 cells at 1 hpi compared to cells infected with *S*. Typhimurium carrying *spvC*. Interestingly, GSDMD-NT levels in the STM-Δ*spvC*/p*spvC* infection group were significantly increased at 3 hpi. The overexpression of the *spvC* gene in the STM-Δ*spvC*/p*spvC* infection group may lead to severe disruption of the intestinal barrier, potentially facilitating *S*. Typhimurium in completing its infectious cycle and enhancing recolonization of the enteroid lumen [39,40]. However, at 16 hpi, there were no significant differences in GSDMD-NT levels among the three strains (Figure 4G,H). This lack of difference may be attributed to the inhibitory effect of *spvC* on GSDMD-related non-pyroptosis pathways or possibly other unknown mechanisms. Notably, we observed that *Il-1β* transcription was progressively upregulated throughout the course of infection, with significantly higher induction in STM-Δ*spvC*-infected mice compared to STM-WT-infected mice from 1 dpi to 5 dpi (Figure 4I). These findings suggest that SpvC suppresses autophagy and pyroptosis in epithelial cells to restrict GSDMD-mediated host defense.

### 3.5. SpvC Arg^90^ Targets SEC23B to Suppress Pyroptosis in Intestinal Epithelial Cells

**Figure 5:**
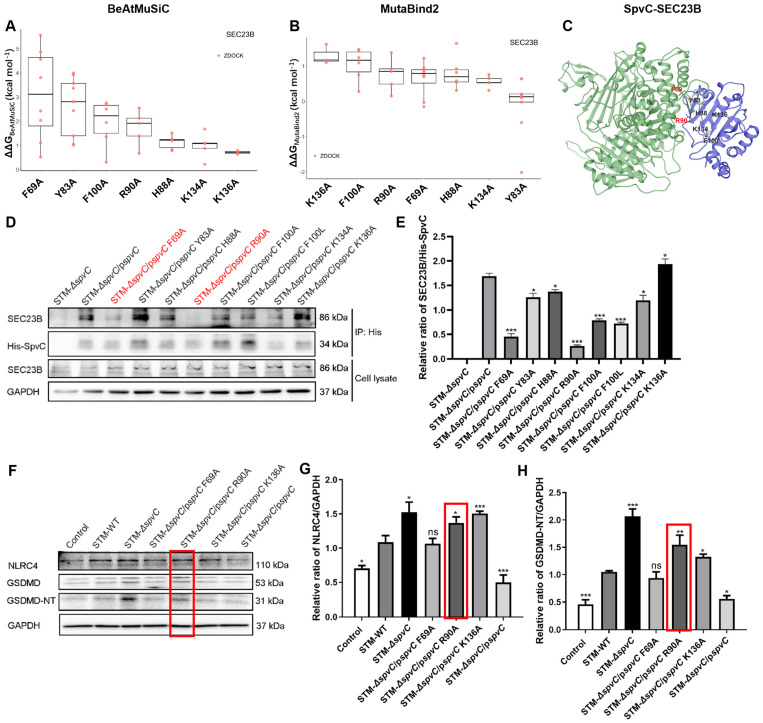
SpvC Arg^90^ targets SEC23B to suppress pyroptosis in intestinal epithelial cells. (**A**,**B**) Changes in binding affinity (∆∆G, kcal mol^−1^) for alanine mutations at the selected interface sites of the top ten highest-scoring conformations from ZDOCK were calculated using BeAtMuSiC (**A**) and MutaBind2 (**B**). (**C**) Three-dimensional model of SpvC-SEC23B generated by ZDOCK, with the 6th docking conformation shown. This conformation was selected based on the predicted ∆∆G values for alanine mutations at the seven key sites and experimental findings. (**D**) STM-Δ*spvC*, STM-Δ*spvC*/p*spvC* and *spvC* site mutant strains were co-cultured with Caco-2 cells at an MOI of 100 for 2 hpi. (**E**) Quantification of Western blotting analysis of (**D**). Caco-2 monolayers were co-cultured with the indicated *S*. Typhimurium strains at an MOI of 100. Red font indicates the key residues of SpvC protein that are responsible for binding to SEC23B. (**F**) NLRC4 and GSDMD/GSDMD-NT were analyzed by Western blotting. Red box indicates the key residues of SpvC protein that affect the protein level of NLRC4 and GSDMD-NT. (**G**,**H**) Quantification of Western blotting analysis on the left. Data were compared by one-way ANOVA. Values are expressed as the means ± SD, and statistically significant differences are indicated. * *p* < 0.05, ** *p* < 0.01, *** *p* < 0.001, ns: not significant. Data were from three biological replicates.

To better define the functional interface between SpvC and SEC23B, an initial selection of critical residues was made based on the ∆∆G using the docking conformations obtained from ZDOCK. This work was followed by further validation and refinement using ∆∆G calculations from ClusPro and HDOCK. For the SpvC-SEC23B interaction, the binding affinity changes for all possible mutations at the interface sites are shown in Figure S3C,D. Based on these results, we selected specific mutants for further experimental investigation. In addition to the BeAtMuSiC predictions, we utilized MutaBind2 for additional validation. The ∆∆G values for all alanine mutations at the interface sites in the SpvC-SEC23B complex are presented in Figure S3E,F. According to both BeAtMuSiC and MutaBind2 predictions, mutations F69A, Y83A, R90A, F100A and K134A significantly reduced the binding affinity of SpvC for SEC23B (Figure 5A,B). Additionally, the F100L and K136A mutation, despite their lower ranking in our predictions, were included in the experimental studies due to their impact on SpvC’s phosphothreonine lyase activity [41]. Lastly, the H88A mutation was randomly selected for experimental testing. Based on these findings, we chose the mutations F69A, Y83A, H88A, R90A, F100A, F100L, K134A and K136A for further experimental validation. The three-dimensional model of the SpvC-SEC23B complex with the seven mutated sites is shown in Figure 5C. We then constructed the site-directed mutant strains as described in the literature [42] and examined their ability to form a complex with SEC23B in *S*. Typhimurium-infected Caco-2 cells. Two mutations, F69A and R90A, largely abolished SpvC’s ability to form a complex with host SEC23B, demonstrating that these residues, F69 and R90, are essential for SpvC’s interaction with SEC23B (Figure 5D,E). Additionally, we assessed the phosphothreonine lyase activity of different SpvC mutants, including the mutant F100L and K136A, to determine whether they disrupted SpvC’s ability to interact with SEC23B. Results indicated that the interface captured in the SpvC-SEC23B complex is slightly influenced by Lys^136^ and partially by Phe^100^ (Figure 5D,E), suggesting that these residues may play a role in an additional function of SpvC beyond its interaction with SEC23B.

To investigate the functional significance of the observed SpvC-SEC23B interface, we co-cultured Caco-2 cells with STM-WT, STM-Δ*spvC*, STM-Δ*spvC*/p*spvC* and its mutants, including F69A, R90A, and K136A. Similar to the *spvC* deletion, the SpvC R90A mutation, but not the F69A mutation, resulted in a significant increase in NLRC4 and GSDMD-NT levels, comparable to those observed in STM-Δ*spvC* groups (Figure 5F–H). In contrast, although SpvC Lys^136^ is not required for complex formation with SEC23B, it partially affected the cleavage of GSDMD-NT compared to the STM-WT and STM-Δ*spvC*/p*spvC* groups (Figure 5F–H). SpvC has been identified as a phosphothreonine lyase that dephosphorylates the MAPK signaling pathway. The MAPK pathway transmits signals from the cell membrane to the nucleus, providing the initial signal for inflammasome transcription. SpvC presumably inhibits the transcription of *Nlrc4* through the MAPK signaling pathway. Furthermore, additional pathways may contribute to SpvC’s inhibitory effect on GSDMD cleavage. Collectively, the above results demonstrate that SpvC Arg^90^ targets SEC23B to suppress pyroptosis in IECs, which may relate to the *S*. Typhimurium systemic infection.

## 4. Discussion

*S*. Typhimurium is a prototype pathogen for studying invasive gut infection in mice [38]. Consistent with prior studies [3,4], our work confirms that the virulence factor SpvC alleviates intestinal inflammation to promote *S*. Typhimurium dissemination. Deletion of the *spvC* gene only partially reduced the bacterial burden in the liver and spleen, indicating that SpvC is a necessary, but not sufficient, factor for the systemic dissemination of *S*. Typhimurium. Our previous work showed *S*. Typhimurium effector SopF regulates PANoptosis of IECs, exacerbating systemic infection [16]. The *S*. Typhimurium effector SpvC can be delivered into host cells through both the salmonella pathogenicity island (SPI)-1 and the SPI-2 type 3 secretion system (T3SS) [3]. The T3SS1 effectors drive *S*. Typhimurium invasion of IECs, while T3SS2 effectors contribute to transmigration into the underlying lamina propria and facilitate the spread to extra-intestinal organs [43]. A key host defense mechanism against enteric pathogens such as *S*. Typhimurium is mediated by GSDMD, which is essential for maintaining mucosal barrier integrity [13]. Our study revealed that the *S*. Typhimurium virulence factor SpvC inhibits macrophage pyroptosis and neutrophil NETosis, thereby subverting GSDMD functions to facilitate bacterial dissemination [12]. Here, we further elucidate the dynamic interplay between SpvC and epithelial-derived GSDMD. Considering the bactericidal activity of GSDMD-NT, we observed increased bacterial colonization in *Gsdmd*^−/−^ mice [44]. The absence of STM-WT in epithelial cells of WT mice underscores the importance of GSDMD-mediated innate immune defense in bacterial clearance. Moreover, the reduced differences in bacterial loads and pathological lesions in the intestines and extra-intestinal organs between groups infected with STM-Δ*spvC* indicated that SpvC counteracts GSDMD-mediated protection during systemic infection. These findings reinforce the crucial role of GSDMD in host defense and illustrate how *S*. Typhimurium employs SpvC to evade immune responses.

The physiological relevance of this antibacterial pathway is evidenced by GSDMD activation in the mouse intestinal tract upon *S*. Typhimurium infection, alongside the evolved strategy of SpvC to counteract this defense. We identified SEC23B, a cellular target of SpvC, as a key factor in GSDMD activation during *S*. Typhimurium infection. SEC23B contains multiple functional domains: the gelsolin-like domain (residues S742/T747) mediates protein transport from the ER to the Golgi [45], while the trunk domain (S186) is phosphorylated by ULK1 to prevent degradation and promote autophagic flux. [35]. The dual functions of SEC23B in cargo transport and autophagy regulation are considered physiologically relevant to the multifaceted roles of GSDMD in host defense.

The intestinal epithelial barrier, including stringent control over cell–cell junctions and fine-tuned replacement of infected epithelial cells, plays crucial roles in orchestrating host–pathogen interactions and maintaining gut homeostasis. We previously found that the *S*. Typhimurium effector SpvB disrupts the intestinal mucosal mechanical barrier to promote bacterial dissemination [46]. Here, we found *S*. Typhimurium utilizes SpvC to specifically target SEC23B in epithelial cells, thereby compromising intestinal mucosal barrier integrity. Importantly, we identified GSDMD as a pivotal protective factor in this context, demonstrating its role in maintaining epithelial barrier homeostasis and restricting bacterial systemic dissemination. This underscores the tug-of-war between host defense mechanisms and bacterial virulence strategies in maintaining gut homeostasis.

Autophagy, a lysosomal degradation pathway that eliminates intracellular pathogens and debris, is also modulated by SpvC [47]. Our previous study validated that *spvC* downregulates inflammasomes in an autophagy-dependent manner [33]. Here, we further show that SpvC targets SEC23B on Arg^90^ to impede autophagy in epithelial cells. Early in infection, the coalescence of SpvC and SEC23B could limit intracellular bacterial viability and replication through a mechanism that relies on autophagosome formation, a process that otherwise facilitates *S*. Typhimurium replication in epithelial cells [48]. This event suppresses NLRC4 activation and GSDMD-mediated pyroptosis, thereby restricting the expulsion of infected IECs to facilitate bacterial dissemination. Later in infection, the elevated levels of NLRC4 in Caco-2 cells infected with *S*. Typhimurium carrying *spvC* likely reflect enhanced epithelial recolonization, creating a reservoir for pathogen replication. It has been previously shown that differences exist in the inflammasome pathways between mouse and human IECs. In contrast to mouse IECs, the NAIP/NLRC4 inflammasome does not play a major role in the pyroptotic response to salmonella in Caco-2 cells and human intestinal epithelial cells. Instead, caspase-4 has been shown to be the primary mediator of this process [49,50]. In our study, while salmonella infection alone did not significantly alter NLRC4 expression in Caco-2 cells, infection with the *spvC*-mutant strain led to an increase in NLRC4 levels. This aligns with SpvC’s function as a phosphothreonine lyase that inactivates MAPKs, which are key upstream regulators of inflammatory signaling that may influence both canonical and non-canonical inflammasome pathways [4]. We demonstrate that interaction between SpvC Arg^90^ and SEC23B inhibits autophagy and pyroptosis in intestinal epithelial cells, thereby disrupting the intestinal mucosal barrier maintained by GSDMD.

**Figure 6:**
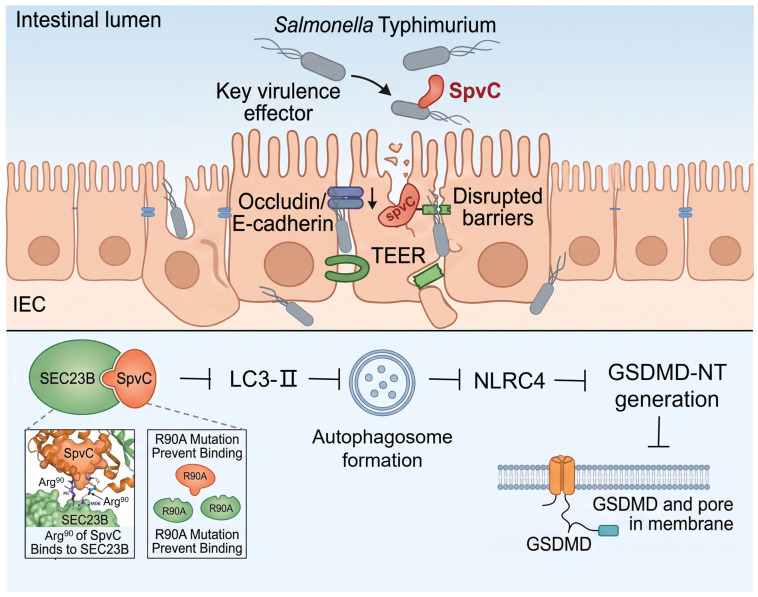
Schematic diagram of salmonella effector SpvC targeting SEC23B of intestinal epithelial cells to resist Gasdermin D-mediated protection against systemic infection. Through direct interaction with host SEC23B, SpvC suppresses both autophagy and pyroptosis in intestinal epithelial cells. Importantly, this SEC23B-dependent pathway enables SpvC to counteract GSDMD-mediated host defense, ultimately leading to intestinal barrier dysfunction and exacerbating systemic bacterial dissemination. This figure was generated with the assistance of Nano Banana, followed by manual editing and refinement by the authors to ensure scientific accuracy.

This study uncovers a unique function of SEC23B in host immunity and provides new insights into the roles of SpvC in salmonella pathogenesis, as summarized in the model depicted in Figure 6. Upon entering IECs, the binding of SpvC Arg^90^ to SEC23B inhibits autophagy and pyroptosis, thereby preventing shedding of infected IECs. These actions facilitate bacterial spread to extra-intestinal organs. These findings reveal a novel pathogen–host conflict between the salmonella effector SpvC and GSDMD in IECs, highlighting the power of bacterial effectors and their genetic mutants in revealing new host defense pathways. As our experiments focused on SEC23B, we shall not rule out the possibility of other SpvC-interacting proteins in host immunity against salmonella dissemination.

## Figures and Tables

**Table 1:** LC-MS/MS screening of SpvC-interacting proteins.

Target Protein Description	Abundance Vector	Abundance SpvC
MYH10, cytoskeleton reorganization	80,400,463	1.94 × 10^9^
MPG, component of membrane	6,302,050	1.92 × 10^8^
ACTC1, actin cytoskeleton	383,983.3	1.58 × 10^8^
DBN1, actin cytoskeleton-organizing protein	806,236.9	1.29 × 10^8^
ABCB10, catalyzes the export of an unknown physiological substrate from the mitochondrial matrix to the cytosol in an ATP-dependent manner	1,717,004	82,282,060
Sec23B, protein processing in endoplasmic reticulum	901,557.6	37,032,032
ATP5PO, mitochondrial membrane ATP synthase	1,386,721	44,950,161
CDK3, Serine/threonine-protein kinase, controls eukaryotic cell cycle	0	12,137,237
NOSIP	596,916	12,072,621
ANAPC2, a cell cycle-regulated E3 ubiquitin ligase	0	11,923,748
AK1, diphosphate kinase activity	0	9,445,620.125
GPN1, small GTPase required for proper nuclear import of RNA polymerase II (RNAPII)	0	5,969,801.063

## Data Availability

The original contributions presented in this study are included in the article/Supplementary Material. Further inquiries can be directed to the corresponding authors.

## Supplementary Materials

The following supporting information can be downloaded at: https://www.mdpi.com/article/10.3390/microorganisms14051148/s1, Table S1: The sources and identifiers of all key reagents and resources; Table S2: Primers used for construction and identification of strains; Table S3: Basic information and structural coverage for SpvC and 30 human protein targets; Figure S1: The bacterial effector SpvC and host GSDMD jointly determine the outcome of *Salmonella* infection; Figure S2: Salmonella effector SpvC overcomes GSDMD-mediated protection from intestinal epithelial barrier of mice; Figure S3: SpvC physically interacts with SEC23B in epithelial cells. References [4,18,51,52] are cited in the Supplementary Materials.

## Abbreviations

The following abbreviations are used in this manuscript:

GSDMD	Gasdermin D
*S*. Typhimurium	*Salmonella* Typhimurium
IECs	intestinal epithelial cells
MAPK	mitogen-activated protein kinase
LC-MS/MS	liquid chromatography–tandem mass spectrometry
SPF	specific pathogen-free
DMEM	Dulbecco’s modified Eagle medium
LB	Luria–Bertani
MOI	multiplicity of infection
PDB	Protein Data Bank
dpi	days post-infection
HE	hematoxylin–eosin
TEM	transmission electron microscope
PTM	post-translational modification
GSDMD-NT	Gasdermin D N-terminal
T3SS	type 3 secretion system
