# Supplementary material for: Salmonella Effector SpvC Targets SEC23B of Intestinal Epithelial Cells to Resist Gasdermin D-Mediated Protection Against Systemic Infection
Source: Microorganisms. 2026 May 19;14(5):1148. doi: 10.3390/microorganisms14051148 (PMC13209872; doi:10.3390/microorganisms14051148)
Supplement: Supplementary file 1 [file microorganisms-14-01148-s001.zip › microorganisms-4315802-supplementary.pdf]

# Salmonella Effector SpvC Targets SEC23B of Intestinal Epithelial Cells to Resist Gasdermin D-Mediated Protection Against Systemic Infection

**Liting Zhou [1,2,3], Yan Yang [1], Li Kang [1,3], Jiayi You [1,3], Ye Wang [1,3], Ailing Xu [1,3], Guangmin Tu [1,3], Rui Huang [1,3], Zhengyu Zhou [4], Minghui Li [1,*] and Shuyan Wu [1,3,*]**

[1] School of Basic Medical Science, Suzhou Medical College of Soochow University, Suzhou 215123, China
[2] Center of Clinical Laboratory, The Fourth Affiliated Hospital of Soochow University, Suzhou 215123, China
[3] MOE Key Laboratory of Geriatric Diseases and Immunology, Suzhou Key Laboratory of Pathogen Bioscience and Anti-Infective Medicine, Suzhou Medical College of Soochow University, Suzhou 215123, China
[4] Laboratory Animal Center, Suzhou Medical College of Soochow University, Suzhou 215123, China;
* Correspondence: minghui.li@suda.edu.cn (M.L.); wushuyan@suda.edu.cn (S.W.)

**Table S1. The sources and identifiers of all key reagents and resources**

| Reagent or resource | Source | Identifier |
|---|---|---|
| **Antibodies** | | |
| Rabbit monoclonal anti-E-Cadherin | Servicebio | Cat# GB12083 |
| Rabbit polyclonal anti-Occludin | Servicebio | Cat# GB111401 |
| Cy3 conjugated Goat Anti-mouse IgG (H+L) | Servicebio | Cat# GB21301 |
| Cy3 conjugated Goat Anti-rabbit IgG (H+L) | Servicebio | Cat# GB21303 |
| Rabbit polyclonal anti-Na, K-ATPase | CST | Cat# 3010 |
| Rabbit polyclonal anti-Occludin | Proteintech | Cat# 13409-1-AP |
| Rabbit polyclonal anti-GAPDH | Boster | Cat# BA2913 |
| Rabbit monoclonal anti-SEC23B | Abcam | Cat# ab245212 |
| Rabbit monoclonal anti-mTOR | CST | Cat# 2983 |
| Rabbit monoclonal anti-FIP200 | CST | Cat# 12436 |
| Mouse monoclonal anti-HA | Beyotime | Cat# AH158 |
| Mouse monoclonal anti-His | Proteintech | Cat# 66005-1-Ig |
| **Bacterial strains** | | |
| *Salmonella* Typhimurium SL1344 | Xu et al., 2019 [1] | N/A |
| *spvC* mutant *Salmonella* (SL1344) | Zuo et al., 2020 [2] | N/A |
| *spvC* mutant *Salmonella* complemented with *spvC* | Zuo et al., 2020 [2] | N/A |
| *spvC* site-directed mutant *Salmonella* strains | This study | N/A |

| | | |
|---|---|---|
| *spvC* mutant *Salmonella* carrying empty pBAD or pBAD for His-tagged SpvC expression | This study | N/A |
| **Chemicals, peptides, and recombinant proteins** | | |
| Luria bertani broth | Hangwei | Cat# B104 |
| Dulbecco’s modified Eagle medium | HyClone | Cat# SH30243.01B |
| Fetal bovine serum | Biological Industries | Cat# 04-001-1ACS |
| Ampicillin | Yeasen | Cat# 60203ES10 |
| L-arabinose | Sigma | Cat# 5328-37-0 |
| Amikacin | Sigma | Cat# 149022-22-0 |
| Disulfiram | MCE | Cat# 97-77-8 |
| Protein A/G PLUS-agarose | Santa cruz | Cat# sc-2003 |
| Anti-HA immunomagnetic beads | Bimake | Cat#B26201 |
| Lipofectamine 3000 | Thermo Fisher Scientific | Cat# L3000008 |
| ExFect transfection reagent | Vazyme | Cat# T101 |
| RIPA lysis buffer | Beyotime | Cat# P0013B |
| Protease and phosphatase inhibitor cocktail | Beyotime | Cat# P1048 |
| Skimmed milk | BBI | Cat# A600669-0250 |
| **Critical commercial assays** | | |
| hematoxylin-eosin | Biosharp | bl700a |
| CF488 TUNEL cell apoptosis detection kit | Servicebio | G1504 |
| Membrane and cytosol protein extraction kit | Beyotime | Cat# P0033 |
| **Experimental models: Cell lines** | | |

| | | |
|---|---|---|
| Human: Caco-2 cells | W. Vallen Graham et al., 2019 [3] | N/A |
| Mouse: J774A.1 cells | Procell Life Science & Technology Co., Ltd | Cat# CL-0370 |
| Human: HeLa cells | ATCC | Cat# CCL-2 |
| Human: HEK293T cells | ATCC | Cat# CRL-3216 |
| **Experimental models: Organisms/strains** | | |
| Mouse: *Gsdmd*$^{-/-}$ C57BL/6 | GemPharmatech Co., Ltd | T010437 |
| **Oligonucleotides** | | |
| SEC23B siRNA target sequence: CACGUUACAUCAACACGGA | Lisa Zeyen et al., 2020 [4] | N/A |
| See Table S3 for full list of primers used in this study | This study | N/A |
| **Recombinant DNA** | | |
| Plasmid: pBAD-SpvC | This study | N/A |
| Plasmid: pEGFP-HA-SpvC | Tsingk Co., Ltd | SH0028002-1 |
| Plasmid: pCDNA3.1-His-SEC23B | This study | N/A |
| **Software and algorithms** | | |
| ImageJ | Launcher broken symmetry software program | https://imagej.nih.gov/ij/ |
| Prism 6 | GraphPad | https://www.graphpad.com |
| CaseViewer | 3DHISTECH Ltd. | https://www.3dhistech.co |

| | | m/ |
|---|---|---|
| **Others** | | |
| Transwells | Labselect | Cat# 14211 |
| Millicell® ERS-2 | Millipore | MERS00002 |
| PVDF membranes | Millipore | IPVH00010 |

**Table S2. Primers used for construction and identification of strains**

| **Name** | **Nucleotide sequence (5' to 3')**[a] |
|---|---|
| F(*Xho* I) | CCTCGAGCCCATAAATAGGCCTAATCT |
| R(*Eco*R I) | GGAATTCCCTCTGTCATCAAACGATAAA |
| F69A-F | TCAGGGATGCGCCAGAGTGGTGCATTTGCTATGAGCCAAGGTTTTCAGCTGAATAACCAT |
| F69A-R | GAAAACCTTGGCTCATAGCAAATGCACCACTCTGGCGCATCCCTGAATAGTCAGGCACAT |
| Y83A-F | TTTCAGCTGAATAACCATGGTGCAGATGTTTTCATCCATGCTCGTCGAGAATCACCTCAG |
| Y83A-R | ACGAGCATGGATGAAAACATCTGCACCATGGTTATTCAGCTGAAAACCTTGGCTCATAGC |
| H88A-F | CCATGGTTACGATGTTTTCATCGCAGCTCGTCGAGAATCACCTCAGTCTCAGGGCAAATT |
| H88A-R | CTGAGGTGATTCTCGACGAGCTGCGATGAAAACATCGTAACCATGGTTATTCAGCTGAAA |
| R90A-F | TTACGATGTTTTCATCCATGCTGCACGAGAATCACCTCAGTCTCAGGGCAAATTTGCCGG |
| R90A-R | CCTGAGACTGAGGTGATTCTCGTGCAGCATGGATGAAAACATCGTAACCATGGTTATTCA |
| F100A-F | ATCACCTCAGTCTCAGGGCAAAGCAGCCGGTGACAAGTTCCACATCAGTGTGCTCAGGGA |
| F100A-R | ATGTGGAACTTGTCACCGGCTGCTTTGCCCTGAGACTGAGGTGATTCTCGACGAGCATGG |
| F100L-F | ATCACCTCAGTCTCAGGGCAAATTGGCCGGTGACAAGTTCCACATCAGTGTGCTCAGGGA |
| F100L-R | ATGTGGAACTTGTCACCGGCCAATTTGCCCTGAGACTGAGGTGATTCTCGACGAGCATGG |

| | |
|---|---|
| K134A-F | TTCAGAGGACAGTCCGGTAGATGCATGGAAAGTGACCGATATGGAGAAGGTCGTTCAACA |
| K134A-R | CTCCATATCGGTCACTTTCCATGCATCTACCGGACTGTCCTCTGAAAACAGCAATCCGGA |
| K136A-F | GACAGTCCGGTAGATAAGTGGGCAGTGACCGATATGGAGAAGGTCGTTCAACAAGCCCGT |
| K136A-R | CGACCTTCTCCATATCGGTCACTGCCCACTTATCTACCGGACTGTCCTCTGAAAACAGCA |

[a] Underling indicates mutant sites. Sequences in the boxes are restriction sites.

**Table S3. Basic information and structural coverage for SpvC and 30 human protein targets**

| Gene | UniProt ID | Full length of protein | PDB ID_ Chain ID | Resolution (Å) | Structure coverage *Pfam domain* |
|---|---|---|---|---|---|
| SpvC | P0A2M9 | 241 | 4HAH_A | 1.80 | 27-241 |
| ATG9A | Q7Z3C6 | 839 | 6WQZ_A | 2.80 | 36-95, 109-535, 539-587 *ATG9 (37-525)* |
| LC3A | Q9H492 | 121 | 5CX3_C | 2.30 | 3-121 |
| FIP200 | Q8TDY2 | 1594 | 6DCE_A | 1.56 | 1494-1591 *ATG11 (1477-1587)* |
| BCL-2 | P10415 | 239 | 5JSN_A | 2.10 | 7-32, 87-209 *BH4 (8-32); Bcl-2 (97-195)* |
| ABCB10 | Q9NRK6 | 738 | 4AYX_A | 2.90 | 154-724 *ABC_membrane (174-438); ABC_tran (511-662)* |
| RPTOR | Q8N122 | 1335 | 6BCX_W | 3.00 | 18-219, 236-686, 806-840, 950-1116, 1125-1292, 1303-1331 *Raptor_N (55-206); HEAT (558-587;604-627); WD40 (1012-1050;1052-1097;1105-* |

| | | | | | |
|---|---|---|---|---|---|
| | | | | | *1151;1154-1194;1200-1240)* |
| mTOR | P42345 | 2549 | 5WBY_A | 3.10 | 1385-1605, 1612-1814, 1867-2436, 2492-2549 *mTOR_dom(854-1024);FAT(1513-1908); FRB_dom(2015-2113); PI3_PI4_kinase(2182-2430); FATC(2517-2549)* |
| BECN1 | Q14457 | 450 | 4DDP_A | 1.55 | 248-264, 269-386, 388-447 *BH3 (105-129); APG6_N (135-261); APG6 (264-445)* |
| DBN1 | Q16643 | 649 | 5Y1Z_A | 2.68 | -1-133 *Cofilin_ADF (14-129)* |
| MYH10 | P35580 | 1976 | 4PD3_A | 2.84 | 8-21, 32-200, 215-365, 367-402, 408-623, 651-783 *Myosin_N (32-76); Myosin_head (87-771); IQ (788-806); Myosin_tail_1 (848-1928)* |
| ATG16 | Q676U5 | 607 | 5NUV_A | 1.55 | 307-607 |

| | | | | | |
|---|---|---|---|---|---|
| | | | | | *ATG16(16-206);WD40(315-349;358-393;400-436;538-562;574-604)* |
| LC3B | Q9GZQ8 | 125 | 2ZJD_C | 1.56 | 1-122 |
| AK1 | P00568 | 194 | 2C95_A | 1.71 | 0-194 |
| ATG12 | O94817 | 140 | 4NAW_A | 2.20 | 53-140 *APG12 (54-140)* |
| ATG101 | Q9BSB4 | 218 | 5C50_A | 1.63 | 1-198 *ATG101 (9-171)* |
| ATG5 | Q9H1Y0 | 275 | 4GDK_B | 2.70 | 3-228, 235-275 |
| ATG13 | O75143 | 517 | 5C50_B | 1.63 | 12-195 *ATG13 (91-197)* |
| ULK1 | O75385 | 1050 | 5CI7_A | 1.74 | 3-179, 181-279 *Pkinase (17-278); ATG1-like_MIT1(875-960);ATG1-like_MIT2(972-1044)* |
| SEC23B | Q15437 | 767 | AlphaFold2 | 95.92 | 1-767 |
| ACTC1 | P68032 | 377 | AlphaFold2 | 97.78 | 1-377 |
| ATG9B | Q674R7 | 924 | AlphaFold2 | 91.57 | 184-676 *ATG9 (185-676)* |
| ZFYVE1 | Q9HBF4 | 777 | AlphaFold2 | 83.31 | 16-777 |
| GPN1 | Q9HCN4 | 374 | AlphaFold2 | 91.37 | 18-299 *ATP_bind_1 (24-262)* |
| PIK3C3 | Q8NEB9 | 887 | AlphaFold2 | 91.25 | 1-413, 473-887 *PI3K_C2 (35-184); PI3Ka(285-521);* |

| | | | | | |
|---|---|---|---|---|---|
| | | | | | *PI3_PI4_kinase (631-831)* |
| ATG14 | Q6ZNE5 | 492 | AlphaFold2 | 92.69 | 43-400 *ATG14 (43-336)* |
| CDK3 | Q00526 | 305 | AlphaFold2 | 92.62 | 1-305 |
| ATP5PO | P48047 | 213 | AlphaFold2 | 88.24 | 24-213 *OSCP (37-209)* |
| WIPI2 | Q9Y4P8 | 454 | AlphaFold2 | 94.36 | 1-279, 318-377 |
| MPG | A2IDA3 | 251 | AlphaFold2 | 97.95 | 68-243 *Pur_DNA_glyco (72-250)* |
| ANAPC2 | Q9UJX6 | 822 | AlphaFold2 | 84.15 | 1-822 |

## Figure S1

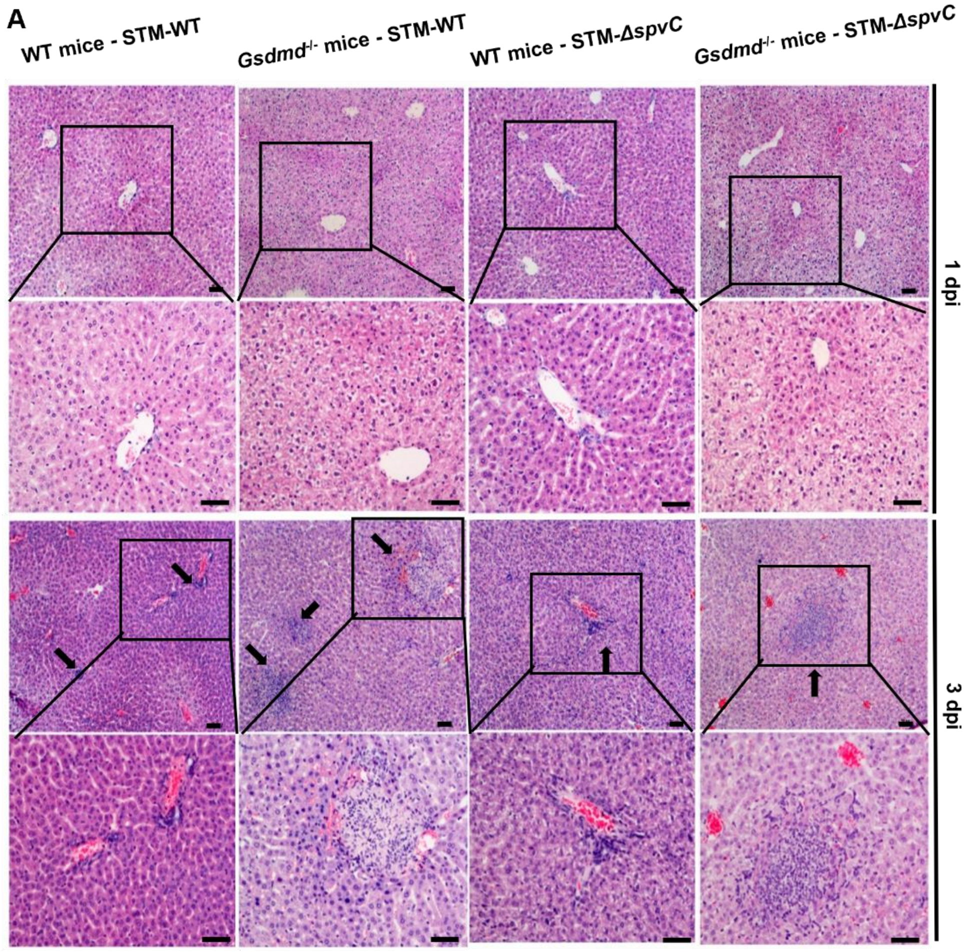

A
WT mice - STM-WT
Gsdmd-/- mice - STM-WT
WT mice - STM-ΔspvC
Gsdmd-/- mice - STM-ΔspvC
1 dpi
3 dpi


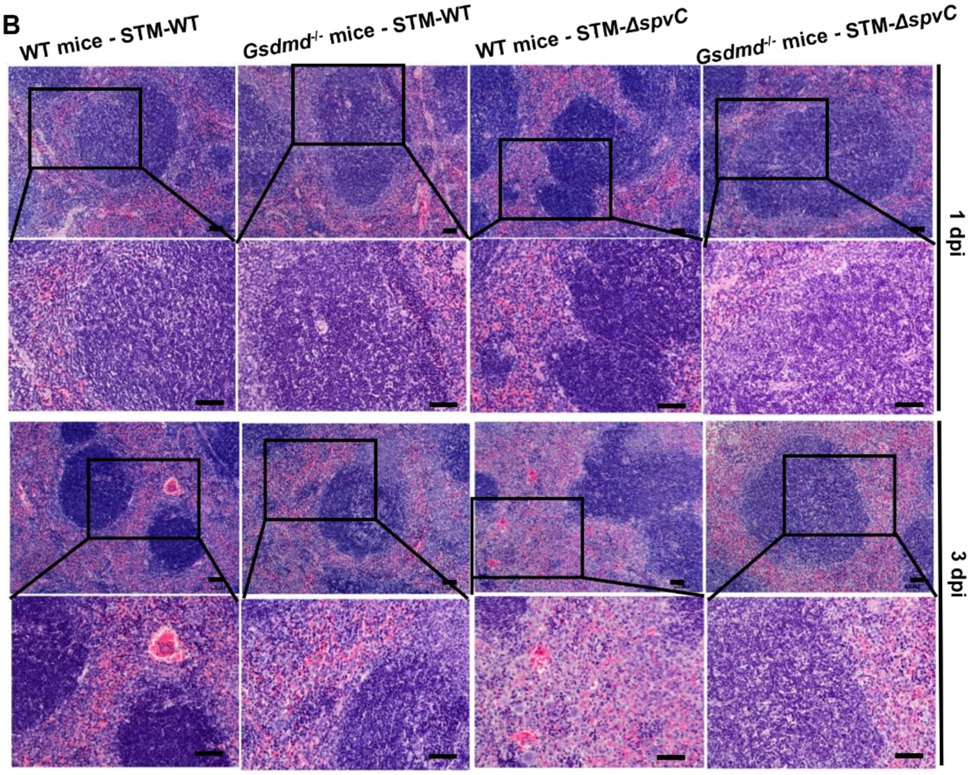

B
WT mice - STM-WT
Gsdmd-/- mice - STM-WT
WT mice - STM-ΔspvC
Gsdmd-/- mice - STM-ΔspvC
1 dpi
3 dpi

**Figure S1. The bacterial effector SpvC and host GSDMD jointly determine the outcome of *Salmonella* infection.** (a and b) C57BL/6J and *Gsdmd*$^{-/-}$ mice were infected orally with $5 \times 10^7$ colony-forming unit (CFU) of either STM-WT or STM-*ΔspvC* after being pretreated with streptomycin. Histopathological analysis of the livers (a) and spleens (b) at 1 dpi and 3 dpi, n = 5. Black arrows indicated infiltration of inflammatory cells. Scale bars, 50 μm. Data were from at least three biological replicates.

## Figure S2

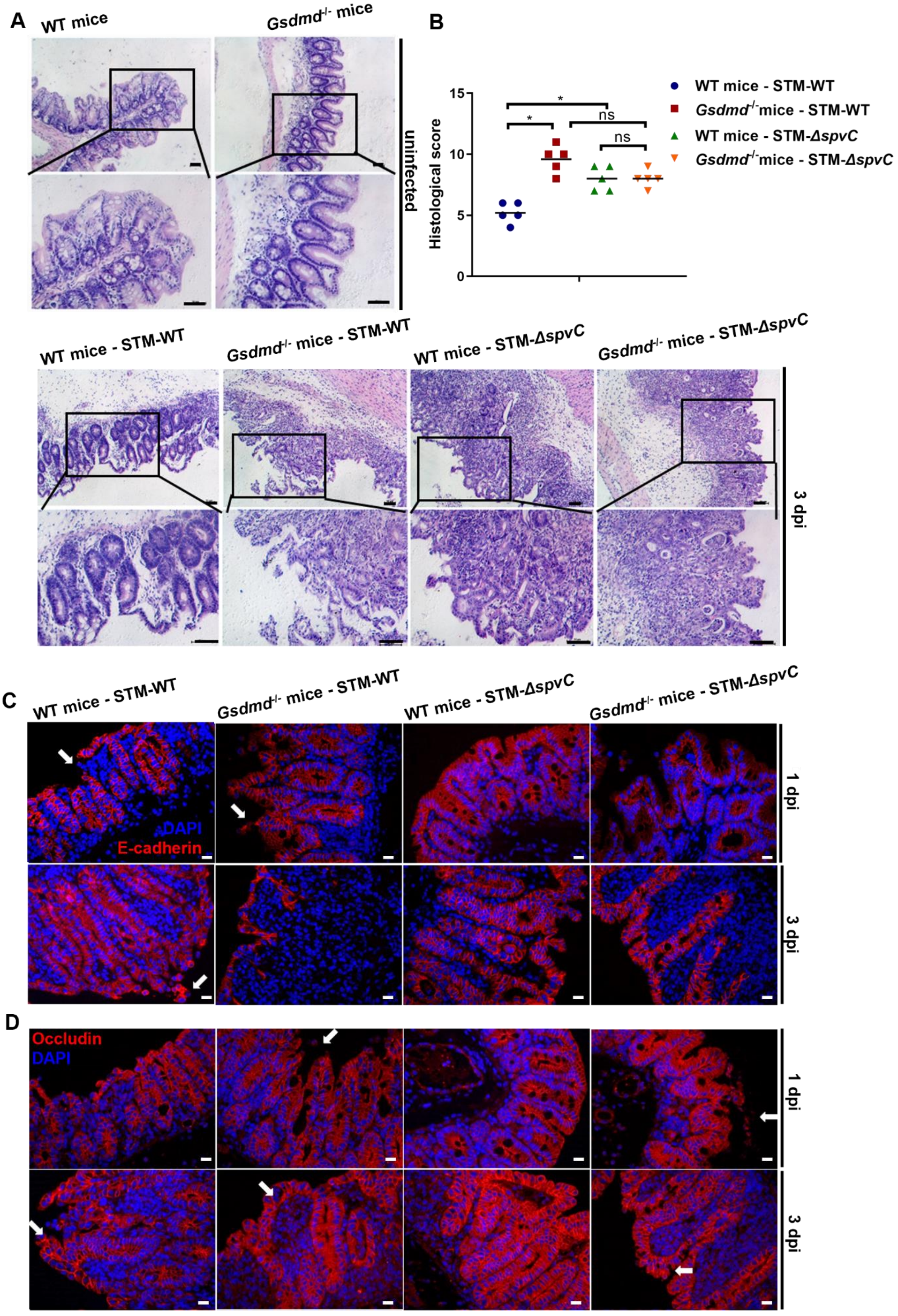
A
WT mice
Gsdmd-/- mice
uninfected
WT mice - STM-WT
Gsdmd-/- mice - STM-WT
WT mice - STM-ΔspvC
Gsdmd-/- mice - STM-ΔspvC
3 dpi
B
Histological score
0
5
10
15
*
*
ns
ns
WT mice - STM-WT
Gsdmd-/- mice - STM-WT
WT mice - STM-ΔspvC
Gsdmd-/- mice - STM-ΔspvC
C
DAPI
E-cadherin
1 dpi
3 dpi
D
Occludin
DAPI
1 dpi
3 dpi

**Figure S2. Salmonella effector SpvC overcomes GSDMD-mediated protection from intestinal epithelial barrier of mice.** C57BL/6J and *Gsdmd*$^{-/-}$ mice were infected orally with $5 \times 10^7$ CFU of either STM-WT or STM-*ΔspvC* after being pretreated with streptomycin. (A) Histopathological analysis of the ceca at 1 dpi and 3 dpi, n = 5. Scale bars, 50 μm. (B) Histopathological score of ceca. Data were from at least three biological replicates. Representative images of immunofluorescence staining for E-cadherin (C) and Occludin (D) on frozen sections of ceca at 1 dpi and 3 dpi, n = 5. White arrows indicated disruption of epithelial barrier. Scale bars, 50 μm.

# Figure S3

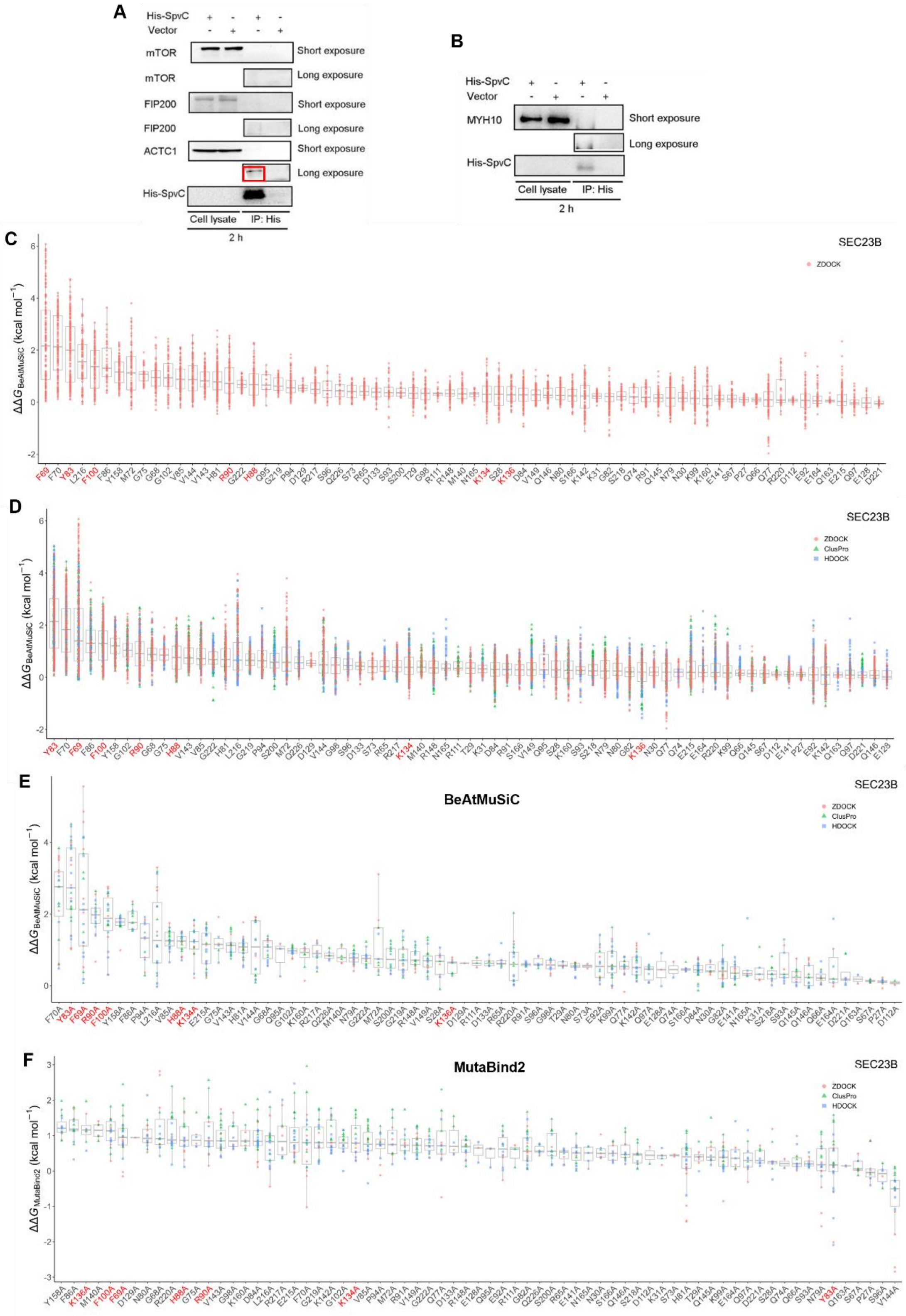

A
His-SpvC
Vector
mTOR
Short exposure
mTOR
Long exposure
FIP200
Short exposure
FIP200
Long exposure
ACTC1
Short exposure
Long exposure
His-SpvC
Cell lysate
IP: His
2 h
B
His-SpvC
Vector
MYH10
Short exposure
Long exposure
His-SpvC
Cell lysate
IP: His
2 h
C
SEC23B
ZDOCK
ΔΔG BeAtMuSiC (kcal mol⁻¹)
D
SEC23B
ZDOCK
ClusPro
HDOCK
ΔΔG BeAtMuSiC (kcal mol⁻¹)
E
BeAtMuSiC
SEC23B
ZDOCK
ClusPro
HDOCK
ΔΔG BeAtMuSiC (kcal mol⁻¹)
F
MutaBind2
SEC23B
ZDOCK
ClusPro
HDOCK
ΔΔG MutaBind2 (kcal mol⁻¹)

**Figure S3. SpvC physically interacts with SEC23B in epithelial cells.** (A and B) Caco-2 cells were infected with STM-*ΔspvC* carrying an empty vector or a vector expressing SpvC: His at an MOI of 100 for 2 hpi. His -tagged SpvC were immunoprecipitated from cell lysates and assessed for their ability to bind SEC23B. Data were from at least three biological replicates. (C and D) Binding affinity changes ($\Delta\Delta G$, kcal mol$^{-1}$) for all possible mutations at the interface sites between SpvC and SEC23B were analyzed using BeAtMuSiC across 30 top-scoring conformations, with 10 conformations generated by each of the tools ZDOCK, ClusPro, and HDOCK. (E and F) Changes in binding affinity for alanine mutations at the interface sites of SpvC-SEC23B were calculated using BeAtMuSiC (E) and MutaBind2 (F) for the same 30 top-scoring conformations from ZDOCK, ClusPro, and HDOCK.
